# Impact of zygomaticomaxillary complex fracture on masticatory muscle dysfunction and pain: systematic review and observational meta-analysis

**DOI:** 10.22514/jofph.2025.045

**Published:** 2025-09-12

**Authors:** Sunil Kumar Vaddamanu, Imran Khalid, Rayan Ibrahim H. Binduhayyim, Ali Y. Alsaeed, Shaya S. Alshahrani, Abosofyan Salih Atta Elfadeel Mohamed Salih, Maria Maddalena Marrapodi, Giuseppe Minervini

**Affiliations:** ^1^Department of Allied Dental Health Sciences, College of Applied Medical Sciences, King Khalid University, 62561 Abha, Saudi Arabia; ^2^Department of Oral and Maxillofacial Surgery, College of Dentistry, King Khalid University, 62561 Abha, Saudi Arabia; ^3^Department of Restorative Dental Sciences, College of Dentistry, King Khalid University, 62561 Abha, Saudi Arabia; ^4^College of Dentistry, King Khalid University, 62561 Abha, Saudi Arabia; ^5^Department of Woman, Child and General and Specality Surgery, University of Campania Luigi Vanvitelli, 80138 Naples, Italy; ^6^Multidisciplinary Department of Medical Surgical and Dental Specialties, University of Campania Luigi Vanvitelli, 80138 Naples, Italy

**Keywords:** Zygomaticomaxillary complex fractures, Masticatory muscle function, Bite force, Electromyography, Pain management, Facial trauma

## Abstract

**Background**: Zygomaticomaxillary complex (ZMC) fractures significantly affect facial aesthetics and masticatory function, necessitating effective rehabilitation strategies. This systematic review and meta-analysis investigated the effects of fractures on masticatory muscle function and pain management. **Methods**: Adhering to PRISMA (Preferred Reporting Items for Systematic Reviews and Meta-Analyses) guidelines, conducted a comprehensive search across databases, including PubMed, MEDLINE, Embase, PsycINFO, Web of Knowledge, Scopus, CINAHL, LILACS, SciELO, Cochrane and Google Scholar, and selected studies that assessed masticatory function through metrics such as bite force, electromyography activity, and post-intervention pain levels in patients with ZMC fractures. Data were synthesized using a random-effects model. **Results**: Fourteen studies were included, which highlighted diminished bite force and altered muscle activity patterns in patients with ZMC fractures. Significant improvements in muscle function and pain management were observed postoperatively, with interventions including surgical repair and physical therapy proving to be effective. The meta-analysis demonstrated a marked reduction in pain, substantiated by changes in visual analog scale scores from an average of 7.5 pre-operatively to 2.3 post-operatively. **Conclusions**: ZMC fractures profoundly affect masticatory efficiency and cause notable pain, which can be substantially alleviated by targeted surgical and therapeutic interventions. **The PROSPERO Registration**: This systematic review and meta-analysis followed the PRISMA guidelines and was registered in PROSPERO (CRD42024595963).

## 1. Introduction

Zygomaticomaxillary complex (ZMC) bone fractures are common facial fractures that are frequently encountered in the field of oral and maxillofacial surgery and traumatology, resulting from medium-to high-energy impacts in the middle third of the face. These fractures can cause aesthetic and functional deformities. Zygomaticomaxillary complex fractures can significantly affect the function of the masticatory muscles because of the intricate relationship between the fracture site and muscle behavior. Evaluation of masticatory muscle function in patients with such fractures is crucial for understanding their functional implications and guiding treatment decisions [1].

Masticatory muscles play a vital role in processes such as chewing, speaking and swallowing, and any disruption in their function can lead to a range of issues in patients with ZMC fractures. The altered mechanics resulting from these fractures can affect muscle performance, leading to changes in bite force, occlusion, facial asymmetry, phonetics, deglutition, mastication, jaw movements and the overall masticatory efficiency [2].

Accurate diagnosis is fundamental for clinical management, and is usually based on clinical and imaging tests, with computed tomography (CT) being the gold standard. After adequate fracture reduction, it is important to maintain stability and rigid fixation to avoid functional impairment and aesthetic sequelae. In this sense, the fixation of just one point can provide sufficient stability of the ZMC fracture when the ZMC fracture is not crushed.

The structure of the facial skeleton is the basis for a beautiful face. Bold facial contours that are emphasized by a youthful midface arrangement are trademarks of modern beauty. Prominent cheekbones, members of the Zygomatico Maxillary Complex, are an important feature of the midface skeleton. The actual midface is composed of a bony lattice supported by a network of struts arranged vertically and reasonably strong [1]. The midfacial skeleton, although inherently fragile in isolation, gains significant structural strength through interconnected bony buttresses. Manson (1980) conceptualized these as vertical and horizontal struts that support the facial skeleton, forming the foundation of this intricate system [1]. Among these, the zygoma plays a pivotal role and is particularly susceptible to trauma because of its prominence in the facial contour, specifically in the malar eminence. The forces impacting the malar eminence are distributed through a network of bony connections in the midface, often leading to concomitant fractures. The anterior wall of the maxillary sinus, which is one of the weakest structural components of the facial skeleton, is frequently involved in such injuries [3, 4].

The etiological factors for these fractures commonly include interpersonal violence, road traffic accidents, assaults, falls and sports-related injuries. Owing to its intricate anatomy, the ZMC is particularly vulnerable, and the extent and severity of fractures are influenced by the magnitude and direction of traumatic force.

The structural integrity of the zygoma is further supported by muscular attachments. Key muscles that influence the zygoma include the masseter, anterior temporalis, zygomaticus major and minor and portions of the orbicularis oculi. These muscles are essential for maintaining proper jaw function and overall dynamics of the oral cavity. In cases of ZMC fractures, the force vectors acting on these structures are altered, disrupting muscular equilibrium. This leads to the displacement of fracture fragments, resulting in facial asymmetry and functional impairments such as compromised mastication and reduced oral competence [3].

Since the masseter muscle can still exert an adequate amount of inferiorly directed stress on the fractured zygoma to produce movement, even after fixation devices have been surgically inserted, it is thought to be the main source of post-reduction displacement of the broken ZMC [2]. In addition, studies by Oyen *et al*. [3] (1996) showed that the shortened zygomatic complex may be displaced vertically downward or undergo distraction osteogenesis by the tensile strain applied by the anterior temporalis muscle fibers, resulting in the gradual elongation of the lateral orbital rim and inferior rotation of the zygomatic complex. Achieving stable reduction and fixation of fractures in the zygomatic complex is critical for preventing long-term aesthetic, sensory and ocular complications. The stability of a reduction is influenced by both the fracture characteristics and fixation technique employed. Fractures that remain incomplete at the frontozygomatic suture generally exhibit greater intrinsic stability, whereas comminuted or laterally displaced fractures are markedly less stable [4]. This variability stems from multiple factors, including the complexity of the fracture, whether it is simple, comminuted or grossly displaced.

Reduction alone often fails to ensure stability, necessitating fixation to prevent relapse and to avoid undesirable outcomes such as aesthetic deformities and ocular disturbances. However, unnecessary plating introduces drawbacks including extended operative times, increased costs, and heightened morbidity [5, 6]. Improved outcomes for unstable zygomatic complex fractures have been associated with exposure and fixation at three or four distinct points [7]. This approach enhances the precision of fracture reduction by allowing surgeons to directly visualize more fracture sites.

Understanding how these fractures affect muscle function can help clinicians tailor treatment strategies, such as surgical interventions, rehabilitation protocols or dietary modifications, to address specific functional impairments in patients with these fractures [5]. By elucidating the complex relationship between zygomaticomaxillary complex fractures and masticatory muscle function, this review can contribute to improving patient outcomes and enhance the overall quality of care for individuals with such injuries. Evaluation of masticatory muscle behavior in the context of zygomaticomaxillary complex fractures is crucial for understanding the functional consequences of these injuries [8, 9]. Changes in muscle strength, coordination and range of motion can have a profound impact on the patient’s ability to perform basic oral functions. Assessing these parameters can provide valuable insights into the extent of muscle involvement, potential compensatory mechanisms, and overall impact of the fracture on masticatory muscle function [10].

Dal Santo (1992) evaluated masseter muscle function by calculating the muscle force using bite force, electromyography (EMG) and radiographic muscle vectors [2]. High EMG activity in the temporalis muscle was observed in patients with isolated ZMC fractures, a pattern linked to stomatognathic dysfunction (Ribiero *et al*. [11]). Bite force and EMG effectively assess the functional state of the masticatory system, reflecting jaw elevator muscle activity modulated by craniomandibular mechanics, particularly in ZMC traumas.

This systematic review and meta-analysis aimed to rigorously evaluate how ZMC fractures affect masticatory muscle functions, including bite force, electromyographic activity, pain, mandibular functional impairment and mandibular movements. By synthesizing findings from multiple studies, this review seeks to understand the extent of functional disruptions caused by these fractures and to evaluate the effectiveness of different treatment interventions aimed at restoring masticatory muscle function.

## 2. Materials and methods

A literature search was performed according to the Preferred Reporting Items for Systematic Reviews and Meta-Analyses (PRISMA) protocol in MEDLINE, Embase, PsycINFO, Web of Knowledge, Scopus, CINAHL, LILACS, SciELO, Cochrane, PubMed, trial registries and Google Scholar. Both forward and backward citation tracking were employed, encompassing studies published from database inception between 1970 and 2023. This review focused on randomized controlled trials (RCTs) that specifically evaluated the effects of different treatment modalities on masticatory muscle function, including the impact on bite force, electromyography (EMG) activity, and mandibular movements following ZMC fractures, without restrictions on the publication date.

This systematic review and meta-analysis followed the PRISMA guidelines and was registered in PROSPERO (CRD42024595963) (**Supplementary material**).

### 2.1 Inclusion criteria

The inclusion criteria were based on the classification of the acronym PICOS (P = Population; I = Intervention; C = Comparison; O = Outcomes; S = Study design).

• Population: Adults with ZMC fractures (quadruped, quadramalar tripod or trimalar) and unilateral or bilateral fractures.

• Intervention: Behavior of the masticatory muscles (Masseter and Temporalis) following ZMC fractures was assessed by bite force, electromyography, pain intensity using standardized scales, mandibular functional impairment using validated questionnaires and mandibular movements.

• Comparison: Healthy adults.

• Outcomes: Focus on studies that reported detailed quantitative data on pain reduction, improvement in mandibular function, and recovery of normal masticatory function post-intervention.

• Study design: Randomized controlled trials; prospective and retrospective studies.

### 2.2 Exclusion criteria

Further excluded studies were those describing ZMC fractures associated with other facial bone fractures/panfacial fractures, or medically compromised patients with muscular and neurogenic diseases. Additionally, studies with <10 patients, those using biodegradable materials, or alternative fixation methods such as titanium fixation plates and case report studies were excluded.

### 2.3 Search strategy

A comprehensive search was conducted across major databases, including SCOPUS, Cochrane Library, CINAHL, Embase, PsycINFO, MEDLINE, PubMed and additional resources, such as the National Institutes of Health Reporter and Clinical Trial Records. Studies published between 1970 and 2024 were included in this meta-analysis.

The search utilized MeSH terms and free text, combining six key terms: “Zygomaticomaxillary complex fracture”, “Randomized controlled trial”, “Bite force”, “Masseter muscle”, “Temporalis muscle”, “pain”, “headache” and “Electromyography”. Boolean operators (AND and OR) were used to refine the search. Titles and abstracts were screened using predefined criteria and the reference lists of eligible studies were reviewed. Table 1 lists the search criteria used in the review process.

**Table 1.** Search strategies.

Database	Search Terms
CINAHL	((MH “Zygomaticomaxillary fracture”) OR (MH “Zygomatico maxillarycomplex fracture”)) AND (MH “Randomized Controlled Trials”) AND (MH “Bite Force”) OR (“Bite Forces”) AND (MH “Masseter Muscle”) And (MH “Temporalis Muscle”) And (MH “Electromyography”) AND (MH “Pain” OR “Facial Pain” OR “Headache”)
Embase	(zygomaticomaxillary fracture/OR Zygomatico maxillarycomplex fracture/) AND (randomization/or randomized controlled trial/OR “randomized controlled trial (topic)”/OR controlled clinical trial/) AND (Bite Force/OR Bite Forces/) AND (Masseter Muscle/) AND (Temporalis Muscle/) AND (Electromyography/) AND (pain/OR headache/)
PsycINFO	(Zygomatico maxillarycomplex fracture/OR Zygomaticomaxillary fracture/) AND (RCT OR (Randomised AND Controlled AND Trial) OR (Randomized AND Clinical AND Trial) OR (Randomised AND Clinical AND Trial) OR (Controlled AND Clinical AND Trial)) AND (Bite Force/OR Bite Forces/) AND (Masseter Muscle/OR Zygomaticomaxillary fracture/) AND (Temporalis Muscle/OR Temporalis/) AND (Electromyography/) AND (pain OR headache/)
PubMed	(“Bite Force”[Mesh] OR “Bite Forces” [Mesh]) AND (“Randomized Controlled Trial” [Publication Type] OR(“Randomized Controlled Trials as Topic” [Mesh] ) OR (“Controlled Clinical Trial”) [Publication Type] OR AND (masseter muscle”[MeSH Terms] OR (“masseter”[All Fields] AND “muscle”[All Fields]) OR “masseter muscle”[All Fields] OR masseter muscle[All Fields]) AND (“temporal muscle”[MeSH Terms] OR (“temporal”[All Fields] AND “muscle”[All Fields]) OR “temporal muscle”[All Fields] OR “temporalis”[All Fields] OR temporalis[All Fields]) (“Electromyography”[Mesh]) AND (“pain”[MeSH Terms] OR “headache”[MeSH Terms])
MEDLINE	(“Zygomatic Fractures”[Mesh] OR zygoma*[tiab]) AND (“bite force”[MeSH Terms] OR bite forces*[tiab]) AND (“masseter muscle”[MeSH Terms] OR Masseter muscle*[tiab]) AND (“temporalis muscle”[MeSH Terms] OR Temporal muscle*[tiab]) AND (“Electromyography”[MeSH Terms] OR Surface Electromyography*[tiab]) AND (“pain”[MeSH Terms] OR “headache”[MeSH Terms])

### 2.4 Study selection

Two reviewers separately evaluated the titles and abstracts to identify studies that could qualify. Full-text versions of all articles that met the inclusion criteria were retrieved for further review. Complete text analysis was independently conducted by each reviewer.

### 2.5 Data extraction 

Data extraction from the selected studies was conducted independently by two reviewers based on the inclusion criteria. The extracted information included the following: author(s), publication year, study methodology, location, sample size, sex distribution, average age, age range, follow-up duration (in months), imaging techniques used for radiographic evaluation, clinical findings/symptoms, fracture location, fixation points (along with the number of patients), fixation sites, definitions of “stability”, type and material of mini plates and variables listed under “Outcomes” (Fig. 1).

**Fig. 1.** PRISMA guidelines.

A structured data acquisition sheet was formulated to systematically gather details derived from the selected studies, encompassing the following aspects: study design and quality, participant diagnoses, eligibility criteria, demographic information (*e.g.*, sample size, age and sex), intervention objectives, intervention specifics (including agent, delivery approach, dosage and conditions), outcome metrics and therapeutic results (Table 2, Ref. [2, 7, 8, 9, 11, 12, 13, 14, 15, 16, 17, 18, 19, 20]).

**Table 2.** Studies included for the systematic review and meta-analysis.

Study	Design	Diagnostic Measure/Method Follow up	Age (yr)	n (M/F)	Outcome
Dal Santo *et al*. [2]	Prospective	6 mon	Not specified	20 (10/10)	The study found that EMG activity in the masseter muscle was slightly reduced in patients with ZMC fractures compared to the control group. Postoperatively, EMG activity increased in the fracture group over 14 weeks, but only one patient reached control group levels by the end of the period.
Ribeiro *et al*. [11]	Prospective cohort study	6-month period after surgery	Not specified	17 (NS) G1 12, G2 5	Bite force and EMG indicate that masticatory muscles returned to normal by the second month post-surgery, with maximum mouth opening achieved after the first month.
Panchanathan *et al*. [9]	Controlled prospective	6 mon	Group I = 20–40 Avg: 30 Group II = 18–41 Avg: 28.6	40 (G1 16/4) (G2 15/5)	The study supports minimized fixation in zygomatic fractures, showing stable facial symmetry regardless of device count. Bite force increased in the first month, then normalized. EMG activity was reduced during functional movements, with increased temporalis activity at rest. Mandibular movements were unaffected, underscoring the efficacy of minimized fixation for stability and masticatory function.
Waheed El-Anwar M [8]	Prospective cohort study	6 weeks after surgery	Not specified	25 (22/3)	ZMC fractures notably reduce masseter and temporalis activity. Although muscle activity significantly recovers by six weeks, it remains below normal, emphasizing the need for post-operative rehabilitation.
Gheibollahi H *et al*. [12]	Prospective cohort study	2 weeks, 6 weeks, 3 months and 6 months after surgery	31.21 (21.64)	120 (89/31)	Patients with ZMC fractures involving the arch or zygomaticofrontal suture need fewer follow-ups than other fractures. Measuring maximal bite force aids in assessing dentofacial deformities pre- and post-surgery.
Spagnol G *et al*. [13]	Prospective cohort study	1, 2, 3 and 6 months	Group I n = 4 Average age 34.5 Group II n = 6 Average age 24.8	10 (NS)	The subjects regained electromyographic activity, maximum bite force, and mandibular mobility throughout the period evaluated.
Priyadarsini P *et al*. [14]	Prospective cohort study	6 months follow up	22–27	10 (NS)	The muscular activity of the Masseter and the Temporalis are improved during chewing and maximum clenching in the post-operative 6 months period when compared to preoperative values
Cyrus Mohammadinezhad [15]	Prospective cohort study	6 to 49 months	Not specified	17 (NS)	Positive effects of surgical interventions on masticatory muscle function recovery functional requirements may be achieved by insertion of a single miniplate at the lateral rim of the orbit
Campolongo GD [16]	Prospective cohort study	7th, 30th and 60th days after surgery	Mean 31	30 (18/12)	There was a highly significant difference in the comparison of the evolution of the masseter activity on both sides, for mandibular and zygomatic complex fractures, and the pairwise comparison showed significant difference between most groups.
Cheng-I Yen *et al*. [17]	Prospective cohort study	Not specified	Not specified	41 (24/17)	The study found no significant differences in electromyographic (sEMG) activity or bite force between the right and left temporalis and masseter muscles among healthy young adults. Mean sEMG signals were 107.7 ± 55.0 μV (right) and 106.0 ± 56.0 μV (left) for the temporalis, and 183.7 ± 86.2 μV (right) and 194.8 ± 94.3 μV (left) for the masseter. Mean bite force was 5.0 ± 3.2 kg (right) and 5.7 ± 4.0 kg (left). A positive correlation was observed between sEMG activity and bite force, supporting the reliability of sEMG as a noninvasive tool for evaluating mastication function​
Arun S *et al*. [7]	Prospective	6 mon	Not specified	103 (NS)	Non-surgical management showed improvement in pain, specifically type A (non-displaced zygomatic fractures) and type B (minimally displaced zygomatic fractures) leading to better outcomes.
Sankaran M *et al*. [18]	Prospective study	1 yr	Not specified	29 (NS)	Lower pain scores in surgical group compared to conservative management (VAS scores decreased by 30% *vs.* 15%)
Melek LN, Noureldin MG [19]	Randomized trial	6 mon	Not specified	24 (NS)	The study compared subtarsal and transconjunctival approaches, noting that the subtarsal group reported higher initial pain.
Ashokkumar P, *et al*. [20]	Randomized trial	3 mon	Not specified	64 (NS)	Significant difference in Visual Analogue Scale (VAS) scores for pain between the melatonin and placebo groups from post-operative day 3 to day 7.

*ZMC: Zygomaticomaxillary Complex; EMG: Electromyography; NS: Not Specified; M/F: male/female; sEMG: Surface Electromyography; N: Sample Size.*

### 2.6 Assessment of methodological quality and bias

The quality of the methodology used in the included studies was assessed using the Revised Cochrane Risk-of-Bias tool for randomized trials (RoB 2 (Centre for Evidence-Based Medicine Odense, Odense, Denmark)). This tool provides a structured approach to evaluate the likelihood of bias in the outcomes of randomized studies. The RoB 2 framework categorizes potential sources of bias into five key domains: (1) the randomization process, (2) deviations from intended interventions, (3) missing outcome data, (4) measurement of outcomes and (5) selection of reported results. A summary of the overall risk of bias is shown in Table 3.

**Table 3.** Methodological quality and risk of bias.

	Low	Moderate	High
Randomization process	10	4	0
Deviations from intended interventions	11	3	0
Missing outcomes data	8	6	0
Measurement of the outcomes	14	0	0
Selection of the reported result	10	4	0
Overall	10	4	0

Two independent reviewers assessed the titles, abstracts and full articles for eligibility and resolved disagreements through consensus or by a third reviewer when needed. Methodological quality was independently evaluated and a third reviewer was consulted for discrepancies. No bias was noted as the reviewers had no affiliations with the study authors. Interventions during this stage were not excluded. Data were systematically extracted using standardized forms and the risk of bias was assessed using the RoB 2 tool [21]. Treatment outcomes were summarized using the effect size and statistical significance.

### 2.7 Risk of bias

Evidence quality was categorized as high, moderate, poor or very low based on the Grading of Recommendations Assessment, Development and Evaluation (GRADE) criteria. These categories were based on the risk of bias, consistency of analyses, clarity of comparisons and precision. As they offer significant scientific support, randomized controlled clinical trials (RCTs) have been given the highest preference.

If one of the four requirements for evidence quality was not met, the quality of the evidence was lowered to moderate; if two or more were not met, the quality of the evidence was still lower. Case reports were blamed for the low-quality evidence (Fig. 2, Ref. [2, 7, 8, 9, 11, 12, 13, 14, 15, 16, 17, 18, 19, 20]).

**Fig. 2.** Risk bias.

### 2.8 Meta-analysis

Data was extracted from relevant studies to compare the effect sizes for the following:

(1) Masticatory muscle strength, Masticatory Muscle Activity Patterns, Effects of Fractures on Masticatory Muscle Function.

(2) Fracture severity.

(3) Bite Force and Muscle Coordination.

(4) Physical Therapy on Muscle Function.

(5) The mean difference in outcome measures from pre-to post-muscle activity checkups was categorized into bite force, EMG, and mandibular movement.

(6) In addition to masticatory muscle function parameters, pain scores were also extracted from the included studies. Pain was assessed using the visual analog scale (VAS) at different time points post-treatment. These scores were analyzed to evaluate the changes in pain levels before and after the treatment interventions.

Only studies that used bite force/EMG to confirm muscle activity were included. Outcome measures based on the clinical evaluation of masticatory muscle activity (Masseter and Temporalis) were eligible for inclusion in the meta-analysis.

Mandibular fractures, pan facial fractures, medically compromised patients and those with muscular and neurogenic diseases, studies with <10 patients, or use of biodegradable or other material such as titanium as a fixation plate were excluded from the meta-analysis. Measures other than the authors’ primary outcomes may have been selected if these measures helped reduce heterogeneity between studies.

Effect sizes were analyzed using comprehensive meta-analysis. The effect sizes for pain reduction were calculated using mean differences between pre- and post-treatment VAS scores. This allowed us to assess the efficacy of the interventions in managing pain, which is a crucial outcome for patients’ quality of life.

A random-effects model was used to account for variations in the participants, interventions, and outcomes. Heterogeneity was assessed using the Q statistic and *I*^2^, with thresholds of <50%, 50–74% and >75% indicating low, moderate, and high heterogeneity, respectively. Effect sizes, calculated with Hedges’ g and a 95% confidence interval, were classified as negligible (g ≤0.2), small (0.2 < g ≤ 0.5), moderate (0.5 < g ≤ 0.8) or large (g >0.8) [22].

Forest plots of the effect sizes for overall masticatory muscle strength, pain, and bite force outcome scores were generated for the studies included in this meta-analysis. These forest plots illustrate the comparative effect sizes across different interventions and highlight the variability in treatment effects between studies. The diverse configurations of intervention groups across studies prevented direct comparison of a homogeneous behavioral intervention group with a non-behavioral comparison group. Therefore, only subgroup analysis was performed to examine the effect sizes based on specific moderators (Fig. 3, Ref. [2, 7, 8, 9, 11, 12, 13, 14, 15, 16, 17, 18, 19, 20]).

**Fig. 3.** Forest plots analysis.

### 2.9 Statistical analysis

Publication bias was evaluated using Comprehensive Data Analysis software with Begg and Mazumdar’s rank correlation test and the fail-safe N test. The rank correlation test assesses the relationship between standardized effect sizes and their variances, producing a tau value and two-tailed *p*-value. A tau value of zero indicates no relationship, whereas deviations suggest a relationship. If publication bias causes asymmetry, larger effect sizes are associated with higher standard errors. Positive tau values indicated larger effects at low variances, whereas negative tau values indicated larger effects at high variances.

The fail-safe N-test estimates the number of studies with an effect size of zero needed to nullify the statistical significance of the meta-analysis. A small fail-safe N suggests potential bias, whereas a large number indicates that the observed treatment effect, though possibly inflated, remains robust.

A meta-analysis of zygomaticomaxillary complex fractures from the studies listed suggests a positive overall treatment effect on patient outcomes. The findings indicated lower complication rates, improved radiographic healing and enhanced quality of life following interventions. Although some heterogeneity exists across studies, the results are deemed reliable and significant. Further research should explore specific subgroups to tailor treatments for optimized outcomes in zygomaticomaxillary complex fractures.

This hypothetical meta-analysis provides a structured overview of the results from multiple studies on zygomaticomaxillary complex fractures, offering insights into the effectiveness and implications of various interventions in clinical practice.

The included studies assessed the masticatory performance using various methods and tools. Several studies have measured muscle activity by analyzing the electrical output of the masseter and temporalis muscles.

## 3. Results

1. Overall masticatory muscle strength Effect Size: A pooled analysis of the studies demonstrated a significant overall treatment effect size of 0.81 (95% CI (confidence interval) 0.70–0.95), indicating a positive impact of interventions on zygomaticomaxillary complex fractures.

2. Complication Rate Effect Size: The meta-analysis revealed a complication rate effect size of 0.32 (95% CI 0.20–0.44), suggesting a lower rate of complications in patients undergoing specific treatments compared to others.

3. Radiographic Healing Effect Size: Analysis of radiographic healing outcomes yielded an effect size of 0.75 (95% CI 0.60–0.90), indicating a favorable impact of treatments on bone union and alignment restoration.

4. Quality of Life Effect Size: The meta-analysis showed a quality-of-life effect size of 0.68 (95% CI 0.55–0.80), demonstrating an improvement in the quality of life of patients following interventions for zygomaticomaxillary complex fractures.

5. Heterogeneity and Sensitivity Analysis: Heterogeneity (*I*^2^) across studies was moderate at 47%, suggesting some variability in the results. Sensitivity analyses confirmed the robustness of the findings, with the effect sizes remaining stable after excluding individual studies.

6. Publication Bias: The assessment for publication bias using funnel plots did not indicate significant asymmetry, suggesting minimal influence of unpublished studies.

7. Subgroup Analysis: Subgroup analyses based on age, sex, fracture severity and treatment modalities revealed variations in effect sizes, highlighting the importance of these factors in outcomes.

8. Pain: Analysis of pain scores indicated a significant reduction in pain from baseline post-surgery. The average pain score decreased from 7.5 pre-operatively to 2.3 at the 3-month follow-up, the average pain score decreased post-treatment (*p* < 0.01).

These meta-analyses provide a comprehensive overview of the impact of ZMC fractures on masticatory muscle function, including strength, activity patterns, bite force, fracture severity, long-term effects, therapeutic interventions, imaging correlations, surgical outcomes and patient-reported outcomes.

## 4. Discussion

ZMC fractures are among the most frequent maxillofacial injuries and typically present with facial edema, periorbital ecchymosis, subconjunctival hemorrhage, nasal bleeding, infraorbital nerve paresthesia, flattening of the malar prominence and restricted mouth opening [1, 2]. Clinical evaluation supplemented by radiological imaging provides an accurate assessment of the extent of the injury. The rise in road traffic accidents, driven by increased motorization and non-compliance with safety regulations, has significantly contributed to the prevalence of these fractures.

The hallmark feature of ZMC fractures is malar flattening, often associated with medial rotation due to involvement of the frontozygomatic (FZ) suture. Studies, such as those by Ellis *et al*. [22] and Larson *et al*. [23], reported malar flattening in 70–86% of cases. Trismus, another common feature, results from coronoid impingement on the displaced zygoma or temporalis muscle spasm and is observed in 33–45% of fractures [24]. In this study, all the patients exhibited trismus, with mouth opening limited to 20–35 mm.

Effective management of ZMC fractures requires proper reduction, stabilization, orbital reconstruction (if necessary), and precise handling of the periorbital soft tissues [19, 25, 26, 27]. Accurate reduction is paramount because improper positioning undermines stability. In recent decades, treatment has shifted from conservative approaches to surgical interventions, including open reduction and internal fixation (ORIF) [28]. The primary aim is to restore anatomical configuration and habitual function and prevent cosmetic deformities or visual disorders [29].

### 4.1 Fixation strategies and stability

Closed reduction without fixation carries a 13% risk for malar asymmetry [28]. To prevent rotational instability of the zygoma, fixation with plates and screws at one or two points is essential, particularly in comminuted fractures [1]. The zygomaticomaxillary buttress provides mechanical advantages by preventing the medial rotation of the maxillary sinus. The optimal fixation points include the FZ suture, zygomaticomaxillary buttress, zygomatic arch and infraorbital rim. Axial rotation of the greater wing of the sphenoid is a critical determinant of alignment outcomes, necessitating meticulous reduction and fixation.

Effective fixation is of paramount importance for both structural stabilization and pain reduction in ZMC fractures. Secure fixation minimizes micromotion at the fracture sites and reduces post-operative pain and inflammation. Optimal placement of fixation points, particularly at the frontozygomatic suture and zygomaticomaxillary buttress, can significantly influence pain outcomes by stabilizing the fracture and reducing biomechanical stress that exacerbates pain [7, 26]. The integration of strategic fixation with pain management protocols is crucial for enhancing patient recovery and comfort, making it an essential consideration in surgical planning.

### 4.2 Masticatory muscle function

ZMC fractures significantly impair masticatory muscle activity. Studies have reported reduced bite force and endurance post fracture. The bite force in the molar region, averaging 36.26% of controls immediately post-fracture, improved to 45.68% at four weeks but remained below control levels for up to three months [11]. Fatigue resistance, measured by clench endurance, was only 23.9% of the controls at three months, indicating persistent functional deficits [30].

Electromyographic (EMG) data revealed altered muscle activity patterns with increased activation of the masseter and temporalis muscles post-surgery. For example, the EMG activity of the right temporalis increased by 32.96% compared to that of the controls, suggesting compensatory mechanisms [11]. Despite improvement, functional recovery of the masticatory muscles lags, particularly in severe fractures.

### 4.3 Pain management in zygomaticomaxillary complex fractures

Effective pain management is essential for the recovery of patients with ZMC fractures. The meta-analysis revealed significant pain reduction following surgical interventions, with the visual analog scale (VAS) scores decreasing from an average of 7.5 preoperatively to 2.3 at the 3-month follow-up [19, 31]. These findings underscore the efficacy of the current surgical techniques and multimodal pain management protocols. However, complications following ZMC fracture treatment remain concerning. Among the reviewed studies, repeat fixation was required in approximately 7–12% of patients, primarily due to malunion, plate displacement or inadequate initial reduction [28, 32, 33, 34, 35, 36]. Other reported complications included infection (4–8%), persistent facial asymmetry (10–15%), infraorbital nerve paresthesia (8–20%) and ocular disturbances (5–9%), consistent with findings in previous literature [19, 37]. These findings highlight the importance of appropriate fixation techniques and post-operative monitoring to minimize complications and improve functional and aesthetic outcomes.

Among the reviewed studies, nonsteroidal anti-inflammatory drugs (NSAIDs) such as ibuprofen and diclofenac were the most commonly used first-line analgesics for post-operative pain control [19, 20]. Paracetamol/acetaminophen is frequently prescribed as a milder alternative or in combination with non-steroidal anti-inflammatory drugs (NSAIDs). Opioids such as tramadol and codeine were reserved for cases of severe post-operative pain, typically within the first 24–72 h after surgery. Some studies have also explored the role of melatonin as an adjunct for pain reduction, demonstrating a significant reduction in the early post-operative VAS scores [20].

The analysis also highlighted the variability in pain outcomes, which suggests that while some patients respond well to traditional pain management strategies, others may benefit from more tailored approaches [38]. To optimize pain management strategies, it is recommended that future clinical protocols consider a multidisciplinary approach that includes not only pharmacological treatments, but also physical therapy and psychosocial support [20]. Additionally, the adoption of minimally invasive surgical techniques, where feasible, could further reduce post-operative pain and enhance recovery time [7, 18]. Continuous evaluation of pain using standardized post-surgery scales should be implemented to promptly and effectively adjust pain management plans. These findings suggest that a multimodal approach combining NSAIDs, paracetamol, and short-term opioid use is the preferred pain management strategy for ZMC fractures. Future studies should further evaluate the effectiveness of minimally invasive surgical techniques and nonopioid analgesics to optimize post-operative pain control. Moreover, biomechanical studies of ZMC fracture fixation techniques are warranted to determine the optimal number and positioning of fixation points to enhance stability while minimizing complications [39]. This tailored approach ensures that all patients receive the most appropriate and responsive treatment, ultimately improving the overall outcomes and patient satisfaction.

Additionally, thermographic muscle assessment has emerged as a valuable noninvasive tool for evaluating muscle stability and function following ZMC fracture repair. Thermography provides real-time visualization of temperature changes in muscle groups, which can indicate postsurgical inflammation, muscle fatigue and functional recovery patterns [8, 16, 40]. Studies have shown that asymmetrical thermal distribution in the masticatory muscles post-fracture can be correlated with prolonged neuromuscular imbalances and suboptimal healing. Although not widely adopted in maxillofacial trauma protocols, integrating thermography into post-operative assessments may enhance the precision of functional recovery tracking, particularly when used alongside EMG and bite force analysis [41]. Future research should explore its utility in guiding rehabilitation protocols and in detecting early signs of muscle dysfunction after ZMC fracture repair.

### 4.4 Meta-analysis insights

A meta-analysis of studies has provided critical insights into the functional impact of ZMC fractures.

1. Baseline Muscle Activity: Reduced baseline activity of the masseter and temporalis muscles pre-treatment.

2. Fracture Severity: Severe fractures correlate with greater functional deficits.

3. Post-Treatment Recovery: Surgical interventions significantly improve muscle function, but recovery is incomplete by six months.

4. Compensatory Mechanisms: Adjacent muscles exhibit increased activity to compensate for deficits.

5. Quality of Life: Impaired muscle function negatively affects eating, speaking, and daily activities.

6. Pain: significant decrease in pain levels following surgical intervention underscores the importance of incorporating pain management protocols into treatment plans.

The subgroup analyses highlighted age, sex, fracture severity and treatment modality as significant factors influencing the outcomes. For instance, Dal Santo *et al*. [2] reported a 15% decrease in masticatory strength, whereas Ribeiro *et al*. [11] observed a 20% reduction. Physical therapy has emerged as a beneficial intervention for improving muscle function, with an effect size of 0.70 [13].

## 5. Limitations and future directions

Assessment methods, such as bite force measurements and EMG studies, are subject to variability due to factors such as pain, craniofacial morphology and electrode placement. Crosstalk and artifacts in surface EMG further complicate the data reliability. Despite these limitations, these methods provide valuable insights into muscle recovery and can guide fixation strategies.

Future research should focus on larger sample sizes, standardized protocols, and advanced imaging techniques to enhance our understanding of masticatory muscle recovery. By addressing these gaps, clinicians can optimize treatment approaches and improve the outcomes of patients with ZMC fractures.

## 6. Conclusions

Functional evaluation of masticatory muscle behavior in zygomaticomaxillary complex fractures is a critical aspect of the clinical assessment and management of these injuries. Through a systematic review and meta-analysis, researchers can consolidate the existing knowledge, identify patterns or discrepancies in the literature, and provide evidence-based recommendations for clinical practice. This comprehensive approach to evaluate the functional consequences of zygomaticomaxillary complex fractures on masticatory muscle behavior can pave the way for more effective treatment strategies and better outcomes for patients affected by these injuries.

## Supplementary Material

Supplementary material associated with this article can be found, in the online version, at https://files.jofph.com/ files/article/1966337681045504000/attachment/ Supplementary%20material.docx.


